# Prostaglandin E2 contributes to *L. braziliensis* survival and therapeutic failure in cutaneous leishmaniasis

**DOI:** 10.1080/22221751.2023.2261565

**Published:** 2023-09-28

**Authors:** Maurício T. Nascimento, Débora L. Viana, Fábio C. Peixoto, Sérgio M. Arruda, Edgar M. Carvalho, Lucas P. Carvalho

**Affiliations:** a Laboratório de Pesquisas Clínicas, LAPEC, Instituto Gonçalo Moniz – Fiocruz, Salvador, Brazil; b Serviço de Imunologia, SIM, Complexo Universitário Professor Edgar Santos, COM-HUPES, Salvador, Brazil; c Programa de Pós-Graduação em Ciências da Saúde, PPgCS, Universidade Federal da Bahia, Salvador, Brazil; d Laboratório Avançado de Saúde Pública, LASP, Instituto Gonçalo Moniz – Fiocruz, Salvador, Brazil; e Instituto de Ciência e Tecnologia em Doenças Tropicais, INCT-DT, Salvador, Brazil

**Keywords:** Cutaneous leishmaniasis, PGE2, therapeutic failure, *L*. *braziliensis*, inflammation

## Abstract

Patients with cutaneous leishmaniasis (CL) present an exacerbated inflammatory response associated with tissue damage and ulcer development. In recent years, higher rates of failure to pentavalent antimoniate therapy have been observed, yet the underlying reason remains poorly understood. We hypothesize that the eicosanoid PGE2 favours the establishment of infection by *L. braziliensis*, which contributes to therapeutic failure. The aim of the present study was to investigate the influence of PGE2 on the survival of *L. braziliensis* in macrophages and rates of therapeutic failure in CL patients. PGE2, an eicosanoid derived from the metabolism of arachidonic acid by the COX-2 enzyme, plays several roles in immune response. We found that increased PGE2 decreases the microbicidal function of macrophages and is associated with disease severity and therapeutic failure. Additionally, the neutralization of COX-2 by NS398, a selective NSAID, increases the ability of macrophages to kill *L. braziliensis* and protects against the pathological inflammatory response. Our data suggest that NS398 may serve as an adjunct treatment for CL patients.

## Introduction

Cutaneous leishmaniasis (CL), an infectious disease caused by parasites of the genus *Leishmania*, results in the appearance of one or more skin ulcers, and represents a major public health problem worldwide [1,2]. In Brazil, Corte de Pedra, a town located in the southeastern region of the state of Bahia, is considered an important area of *L. braziliensis* transmission, with an alarming incidence of CL reaching 1000 cases/year in recent years [3]. In areas endemic for *L. braziliensis*, Glucantime^®^ is the drug of first choice, yet effectiveness varies as around 70% of patients in the pre-ulcerative phase exhibit therapeutic failure [4,5]. The development of specific and effective treatments require a more robust understanding of the pathogenesis of CL caused by *L. braziliensis*.

The course of CL is characterized by an intense inflammatory reaction with a marked predominance of lymphocytes and mononuclear phagocytes [1,6]. Although the inflammatory response is extremely necessary to control parasite replication, the exaggerated production of inflammatory cytokines can cause tissue damage and contribute to the emergence of ulcers that typify this disease [7]. Mononuclear phagocytes, one of the main cell types harbouring *Leishmania*, produce inflammatory cytokines, such as TNF and IL-1β, which participate in tissue damage and the development of ulcers [8–11].

PGE2 is an inflammatory eicosanoid derived from the metabolism of arachidonic acid (AA) by two isoforms of cyclooxygenase, the constitutive isoform COX-1 and COX-2, the induced isoform [12], which are produced by several cell types (e.g. neutrophils and macrophages). The activity of PGE2 in innate immune responses is critically dependent on interaction with one of its four prostanoid receptors which are coupled to G protein (EP1-4). The activation of the EP1 receptor by PGE2 increases intracellular calcium accumulation, while the activation of EP2 and EP4 receptors induces the production of adenylate cyclase and cAMP; in turn, the activation of EP3 decreases cAMP formation [13–15]. Increases in PGE2 and cAMP leads to the activation of protein kinases and decreased microbicidal activity of mononuclear phagocytes [16,17]. The aim of the present study was to investigate the role of PGE2 in *L. braziliensis* infection and elucidate its participation in the exacerbated inflammatory response observed in CL patients.

Our results demonstrate the increased expression of genes *PTGS2, PTGER1, PTGER2* and *PTGER4* in active CL lesions. In addition, the expression of PTGS2 and COX-2 protein was further associated with parasite load, disease severity and failure to therapy. Finally, we found high levels of PGE2 in the cells of CL lesions, which positively correlated with higher numbers of lesions. The neutralization of COX-2 in the cells obtained from lesions decreased the levels of TNF, IL-1β and IL-10 *in vitro*. Surprisingly, the neutralization of COX-2 was found to increase the killing of *L. braziliensis* by macrophages.

## Materials and methods

### Ethics statement

The present study was approved by the Institutional Review Board of the Federal University of the State of Bahia (CEP-UFBA, protocol number 2.471.185) and the Brazilian National Commission for Ethics in Research (CONEP protocol number 2.512.434). All included individuals were adult volunteers who provided written informed consent. All procedures described herein were conducted in accordance with the Declaration of Helsinki.

### Patient sample

Patients with CL were diagnosed based on the presence of a typical skin ulcer associated with PCR positive for *L. braziliensis* DNA as well as a positive intradermal skin test for *Leishmania*. All patients were treated with Glucantime^®^ (Sanofi-Aventis) at the dosage of 20 mg/kg/day intravenously for 20 days. At day 90, patients were reevaluated for lesion resolution. Cure was defined as total re-epithelialization of cutaneous lesions. Patients with lesions that remained active at day 90 were considered to have failed treatment and were submitted to a susbsequent round of Glucantime®. All immunological analyses were performed prior to the initiation of drug therapy.

### *In silico* analysis

Data samples were downloaded from GSE127831 [18] and labelled according to the informed metadata (21 samples infected by *L. braziliensis* before treatment, and 7 uninfected endemic controls), as well as transcripts of *L. braziliensis* sequenced by RNA-seq. Heat map and fold change illustrations were constructed using Multi Experiment Viewer (MeV).

### *In situ* analysis

Skin biopsies (11 samples infected by *L. braziliensis* before treatment and 8 uninfected endemic controls) were fixed in 4% paraformaldehyde and embedded in paraffin. Embedded tissue was cut into 2-μm thick sections, deparaffinized, and rehydrated. Antigen retrieval was performed using 1:100 Trilogy™ solution (Cell Marque) at 96°C. Peroxidase activity was blocked with 3% hydrogen peroxide for 10 min, while nonspecific antibody binding was blocked by the addition of serum-free protein (Dako) for 10 min. Slides were incubated for 2 h with the following antibodies: Rabbit polyclonal to COX2/Cyclooxygenase 2 (1:250) (ABCAM); Peroxidase Kit and Rabbit mouse/horseradish peroxidase KP500 (Diagnostic BioSystems, Pleasanton, CA); Additionally, histological sections were stained with hematoxylin and eosin (H&E) for parasite load quantification. In each field, the number of positive cells and amastigotes were quantified using the semiautomatic counting tool of ImageJ 1.48v software (National Institutes of Health, Bethesda, MD).

### Parasite cultures

L. braziliensis (MHOM/BR/LTCP11245) was isolated from a skin lesion of a CL patient, and confirmed as L. braziliensis by multilocus enzyme electrophoresis [19]. The parasites selected for this study were not previously passaged in liquid culture medium. Following selection, parasites were expanded in Schneider's medium (Sigma) supplemented with 20% heat-inactivated fetal bovine serum, penicillin 100 UI/mL, and streptomycin 100 µg/mL (Gibco).

### Human macrophage cultures and *L. braziliensis* infection

Monocyte-derived macrophages from CL patients were prepared following a previously described method [8] to yield macrophages 99% characterized by flow cytometry as CD14-positive, CD3-negative and CD19-negative. After differentiation, cells were infected with stationary phase *L. braziliensis* promastigotes at a 5:1 ratio for 2 h; uninfected macrophages were used as controls. Following incubation, cells were infected with L. braziliensis and cultured in the presence or absence of NS398 (100 µM) (Sigma, St. Louis, MO), PGE2 10 ng/mL or rIL-10 (0.5, 1, 2 ng/mL) (R&D Systems, Minneapolis, MN USA) for 2, 48 or 72 h. After each time point, the infection rate and parasitic load were evaluated by three different observers using microscopy.

### Skin fragment cultures

Biopsies of the active CL lesion and healthy skin were performed using a 4 mm punch. Fragments were dispensed in tubes containing 1 mL of RPMI-1640 (Gibco) supplemented with 10% FBS (Gibco), 100UI /mL penicillin, and 100 µg/mL streptomycin and incubated at 37°C, 5% CO_2_ for 72 h in the presence or absence of NS398 100 µM (Sigma, St. Louis, MO).

### Determination of cytokine and eicosanoid levels

Levels of PGE2, IL-6, IL-1β, TNF and IL-10 (R&D Systems, Minneapolis, MN USA) were evaluated in culture supernatants by competitive or sandwich ELISA technique. All assays were performed following the manufacturer's protocol. For PGE2, a competitive ELISA was performed, and for TNF, IL-6, IL-1β and IL-10, sandwich ELISA was performed.

### Statistical analysis

The Mann–Whitney test and/or unpaired *t*-test were used for comparisons between two independent continuous variables. Wilcoxon's *U* test and/or the Paired *t*-test were used for continuous dependent variables, while log-rank (Mantel–Cox) testing was used for survival analysis and Spearman's and/or Pearson's tests were used to establish correlations. All analyses were performed using GraphPad Prism v8, and results presenting a *P*-value <0.05 were considered statistically significant.

## Results

**Figure 1.**
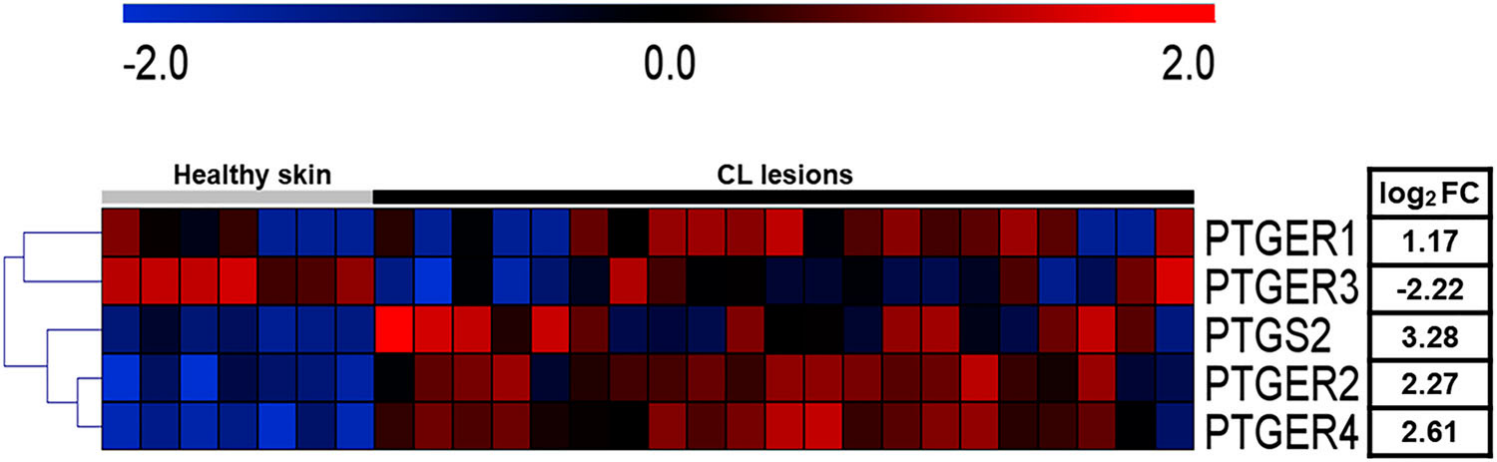
High expression of PGE2 pathway components is observed in active CL lesions. Unbiased RNAseq was performed on skin samples from 7 healthy subjects and lesions from 21 CL patients. Heatmap columns and rows representative of individuals and genes, respectively. Heatmap colour reflects raw z-scores of gene abundance across samples.

To better understand the components of the PGE2 pathway present at the sites of CL lesions, previously published gene expression data was re-analysed [18]. The new analysis revealed increased expression of *PTGS2, PTGER1, PTGER2* and *PTGER4,* genes involved in the PGE2 pathway, in CL lesions, whereas expression levels of *PTGER3* were decreased in comparison to healthy skin (Figure 1A). These results suggest that elevated expression of COX-2 (*PTGS2*) and EP1, EP2 and EP4 receptors (*PTGER1*, *PTGER2* and *PTGER4*) may be a trigger for *L. braziliensis* survival and increased inflammatory response.

**Figure 2.**
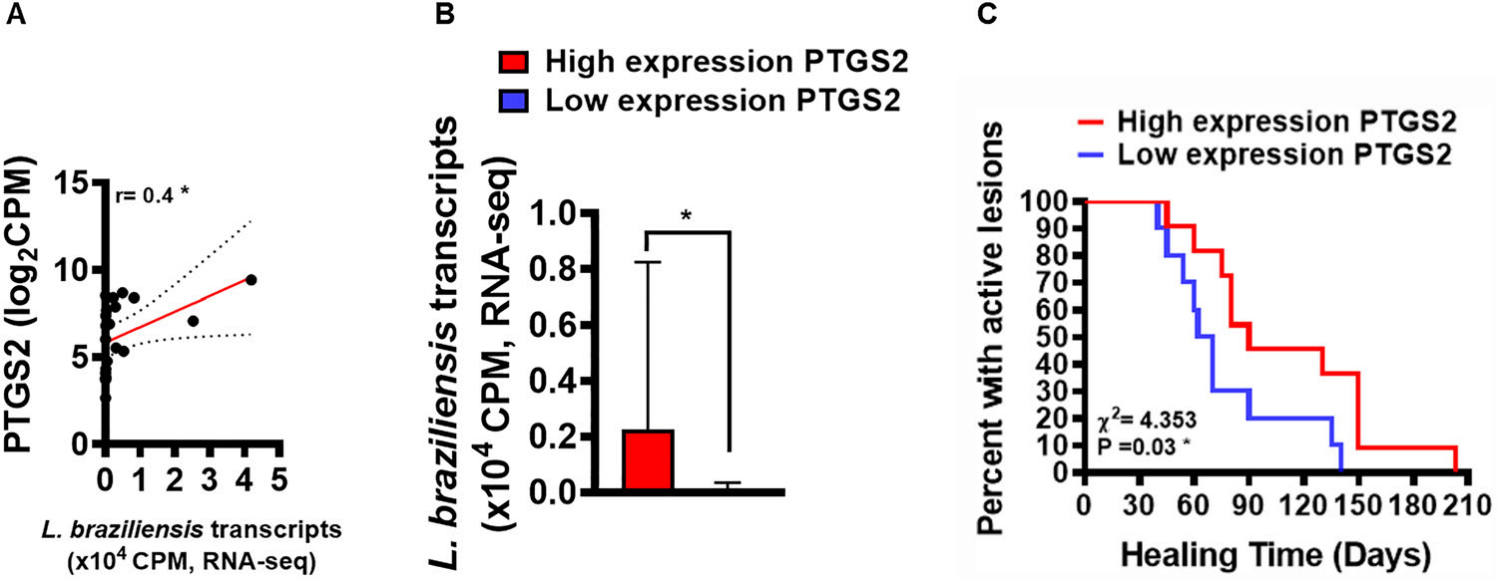
Increased PTGS2 gene expression at the lesion site is associated with parasite load and clinical outcome. RNAseq data (from 21 lesions) was used to investigate correlations between gene *PTGS2* and number of *L. braziliensis* transcripts. The mean expression of *PTGS2* was used as a cut-off point to determine high or low expression. *L. braziliensis* transcript number presented as median and interquartile range, with red bar indicating the group with high expression from *PTGS2* and blue bar low expression. Statistical analysis performed using Pearson's testing for correlations (A); the Mann–Whitney test was used for comparing parasite load in groups with high and low PTGS2 expression (B); Kaplan-Meier survival analysis of groups with respect to therapeutic failure (C) *P < 0.05.

PGE2 has been described as an important eicosanoid related to *Leishmania* infection susceptibility in murine models [17,20]. To evaluate whether increased *PTGS2* gene expression at lesion sites of patients with CL could be involved in parasitic load and clinical outcome, we attempted to establish correlations between the number of *L. braziliensis* transcripts found in active CL lesions and *PTGS2* gene expression. Next, utilizing mean *PTGS2* expression as a cut-off point, we distinguished between patients with high or low expression, and then analysed respective parasite loads and patient clinical outcomes. A positive correlation was observed between *PTGS2* gene transcripts and parasitic load (Figure 2A). In addition, we found that patients with high *PTGS2* gene expression exhibited higher parasitic load than those with lower gene expression (Figure 2B). To test whether increases in *PTGS2* gene expression could be associated with therapeutic failure, a Kaplan-Meier survival analysis was performed in both high expression and low expression groups, revealing an association between therapeutic failure and increased *PTGS2* gene expression (Figure 2C). These results reinforce the hypothesis that increased *PTGS2* gene expression at the lesion site may be an important host factor involved in the establishment of *L. braziliensis* infection that may consequently lead to therapeutic failure.

**Figure 3.**
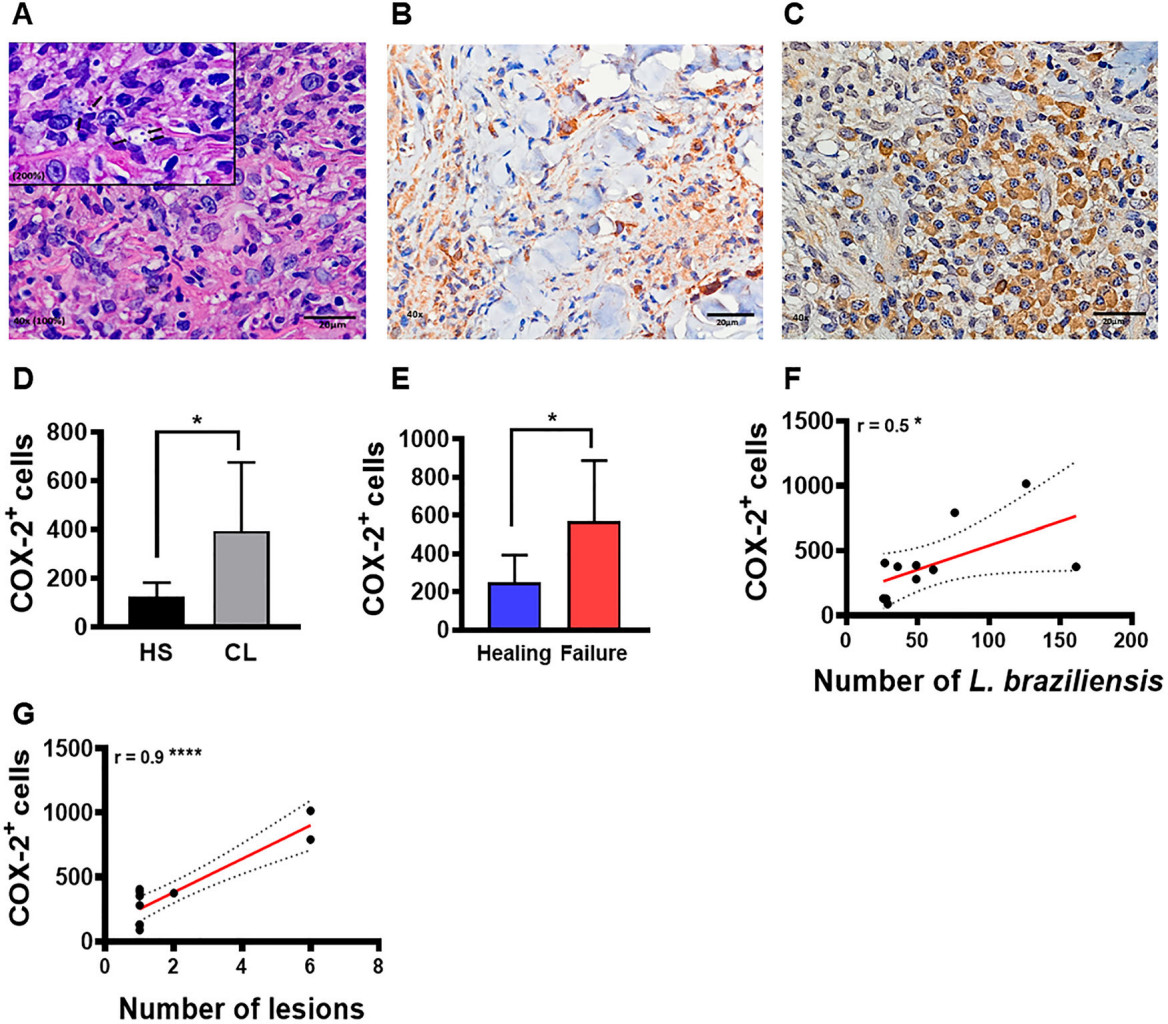
High frequency of COX-2^+^ cells at the lesion site is associated with parasite load, disease severity and therapeutic failure. Lesion biopsies from CL patients (*n* = 11) and skin samples from healthy subjects (*n* = 8) were obtained using a 4 mm punch. Black arrows indicate the presence of *L. braziliensis* amastigotes in CL lesions (A), cells immunostained with polyclonal antibody anti-COX2 in healthy skin (B) and CL lesions (C), respectively. COX-2^+^ cell frequency presented as mean and standard deviation: black bar indicates healthy skin while grey bar represents CL lesions (D). Blue bar indicates patients that evolved to cure, while the red bar patients those that failed therapy (E) Correlation between number of amastigotes and frequency of COX-2^+^ cells (F), and correlation between number of lesions and frequency of COX-2^+^ cells (G). Statistical analysis performed using the Unpaired t-test to compare COX-2^+^ frequency, with Pearson's testing used for correlation analysis; *P < 0.05, ****P < 0.0001.

To confirm that increased COX-2 expression at the lesion site may be involved in *L. braziliensis* survival, we evaluated the relationship between COX-2^+^ cell frequency at the lesion site and parasite load as well as clinical outcome. A high frequency of COX-2^+^ cells were found at lesion sites compared to healthy skin (Figure 3D). In addition, we observed that patients who failed Sb^v^ therapy presented higher frequencies of COX-2^+^ cells (compared to those who exhibited lesion healing) (Figure 3E). Finally, we identified that a higher frequency of COX-2^+^ cells positively correlated with amastigote numbers (Figure 3F) and quantity of lesions (Figure 3G).

**Figure 4.**
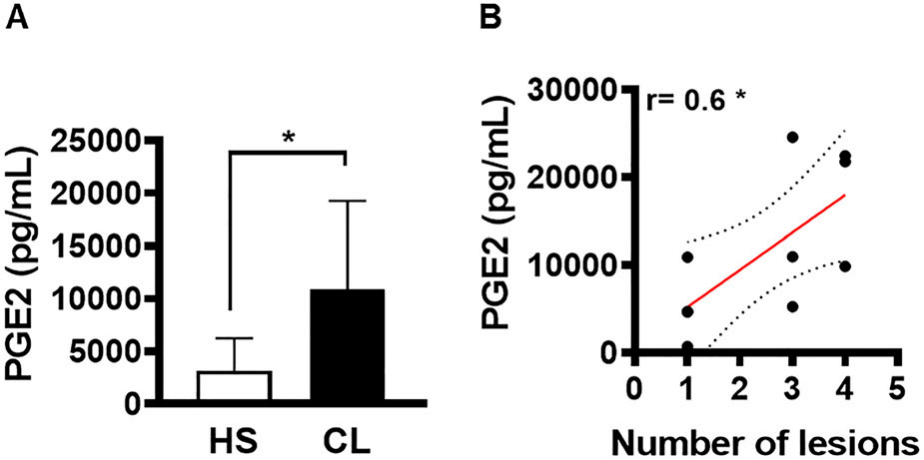
PGE2 production in active lesions correlates with disease severity. Lesion biopsies from CL patients (*n* = 11) and skin samples from healthy subjects (*n* = 8) were obtained using a 4 mm punch and cultured for 72 h. PGE2 levels were determined in culture supernatants by ELISA. (A) Levels of PGE2 presented as mean and standard deviation. (B) Correlation between number of lesions and PGE2 levels. Statistical analysis performed using the Unpaired t-test to compare PGE2 levels, while Pearson's testing was used for correlation analysis; *P < 0.05.

It was previously shown that patients who fail therapy present high parasitic load [18]. In an attempt to identify the mechanisms that favour the establishment of infection, we evaluated the ability of cells residing in active CL lesions to produce PGE2. First, we investigated whether PGE2 was produced at active lesion sites of CL patients. Compared to healthy skin, we observed high levels of PGE2 production by cells residing in CL lesions (Figure 4A). Next, we endeavored to determine whether the high production of PGE2 was associated with disease severity, and found a positive correlation between PGE2 levels and the number of lesions (Figure 4B).

**Figure 5.**
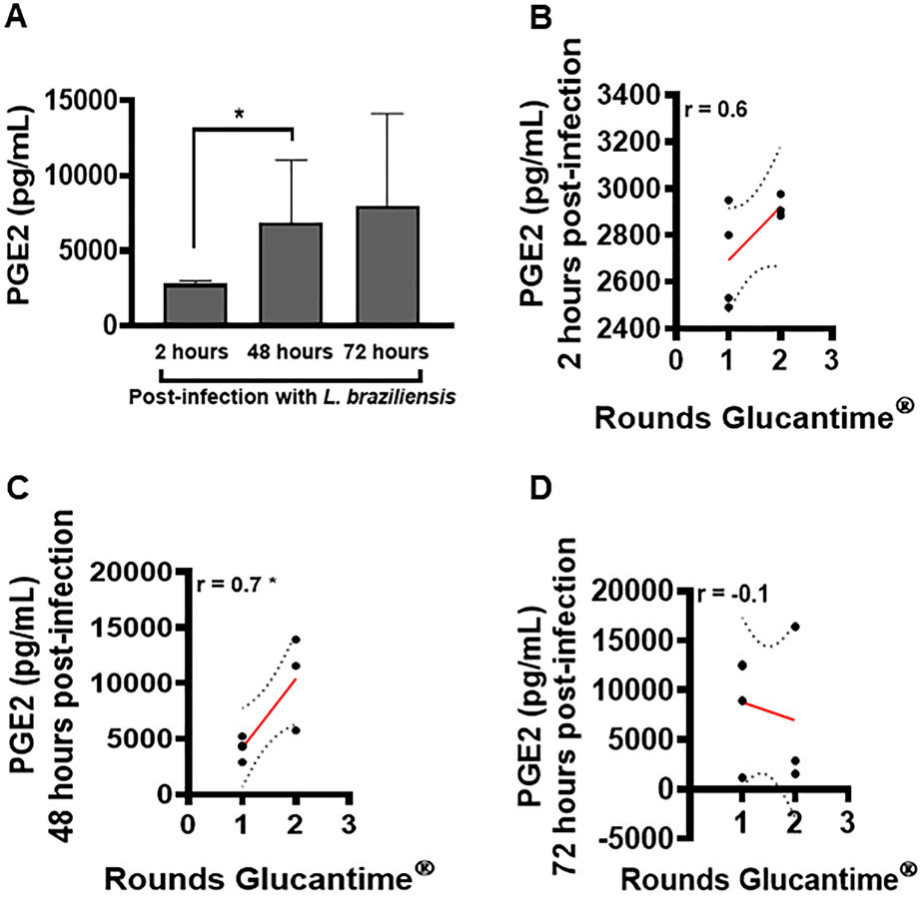
High levels of PGE2 in macrophages infected with *L. braziliensis* correlate with treatment duration. Monocyte-derived macrophages obtained from CL patients (*n* = 7) were infected with stationary-phase *L. braziliensis* (5:1) and cultured for 2, 48 or 72 h. PGE2 levels were determined in culture supernatants by ELISA. (A) PGE2 levels presented as mean and standard deviation. (B) Correlation between rounds of Glucantime^®^ and PGE2 levels. Statistical analysis performed using the Unpaired t-test to compare the PGE2 levels, while Pearson's testing was used for correlation analysis between lesion number and PGE2 levels; *P < 0.05.

An elevated frequency of macrophages has been identified in the inflammatory infiltrate of CL lesions [6]. These cells are extremely important for the effective control of parasitic load [8]. We investigated the ability of these cells to produce PGE2 following *L. braziliensis* infection, as well as the role of this eicosanoid in the pathogenesis of CL. We identified low PGE2 production by macrophages at 2 h after *L. braziliensis* infection, which increased significantly at 48 h post-infection; however, the difference observed at 72 h post-infection was not statistically significant compared to 48 h (Figure 5A). In addition, at 48 h post-infection, increased levels of PGE2 were found to positively correlate with the number of rounds of Glucantime^®^ (Figure 5C).

**Figure 6.**
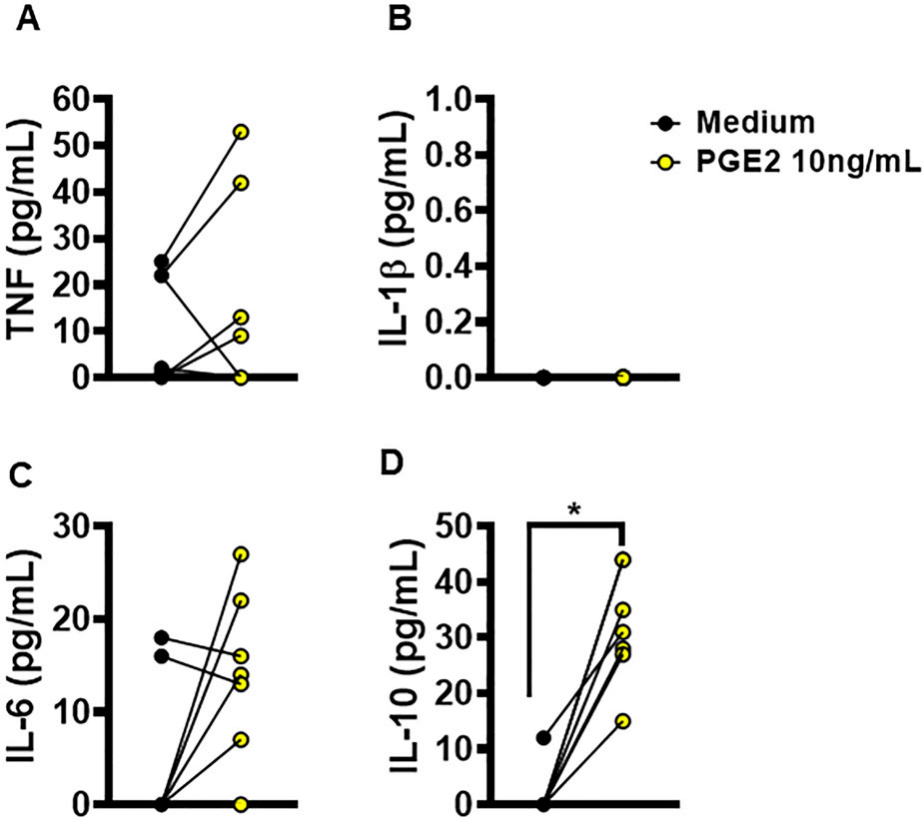
Ability of PGE2 to alter macrophages cytokine production. Monocyte-derived macrophages from HS (*n* = 5) were cultured in the presence or absence of PGE2 (10 ng/mL) for 72 h. The levels of (A) TNF, (B) IL-1β, (C) IL-6 and (D) IL-10 were determined in culture supernatants by ELISA. Statistical analysis performed using the Wilcoxon test; *P < 0.05.

Previous studies have reported that increased PGE2 induces the production of IL-1β in macrophages [21,22]. To test whether PGE2 influences the production of these cytokines, we enriched cultures of uninfected macrophages from CL patients with PGE2 to evaluate levels of TNF, IL-1β, IL-6 and IL-10. Interestingly, we observed that the enrichment of macrophage cultures with PGE2 did not affect inflammatory cytokine production (Figure 6A–C); however, by way of an unclear mechanism, PGE2 did induce the production of IL-10 (Figure 6D). To investigate whether the amount of IL-10 induced by PGE2 had an effect on the ability of macrophages to kill *Leishmania,* we then infected macrophages from CL patients and enriched the cultures with increasing doses of rIL-10. We found that increases in IL-10 impeded the ability of macrophages to kill *L. braziliensis*, thereby allowing parasites to proliferate (supplementary Figure 1). It follows that the inhibition of PGE2 synthesis may represent a promising strategy for in the control of inflammatory response and infection by *L. braziliensis in vitro*.

**Figure 7.**
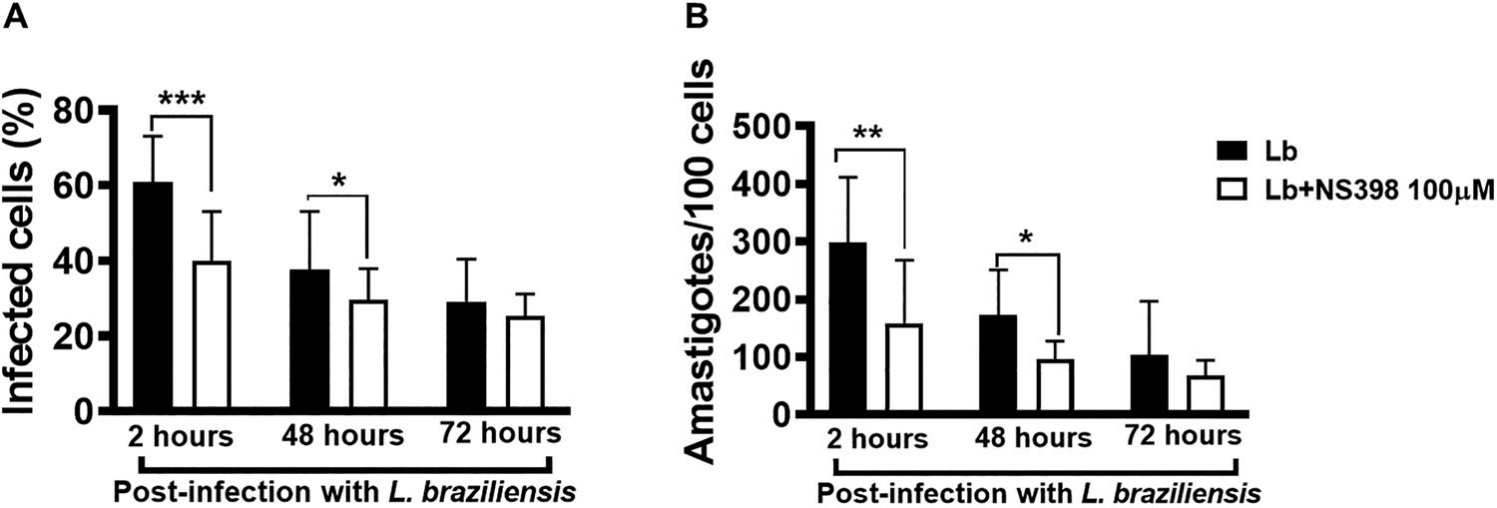
Neutralization of COX-2 increases *L. braziliensis* killing by macrophages obtained from CL patients. Monocyte-derived macrophages from CL patients (*n* = 7) were infected with stationary-phase *L. braziliensis* (5:1) and cultured in the presence or absence of NS398 (100 µM), a selective COX-2 inhibitor, for 2, 48 or 72 h. (A) Frequency of infected cells. (B) Number of amastigotes per 100 macrophages. Results presented as mean and standard deviation, with black bars indicating macrophages infected with *L. braziliensis,* and white bars macrophages infected with *L. braziliensis* treated with NS398. Statistical analysis performed using the Paired t-test; *P < 0.05, **P < 0.005, ***P < 0.001.

**Figure 8.**
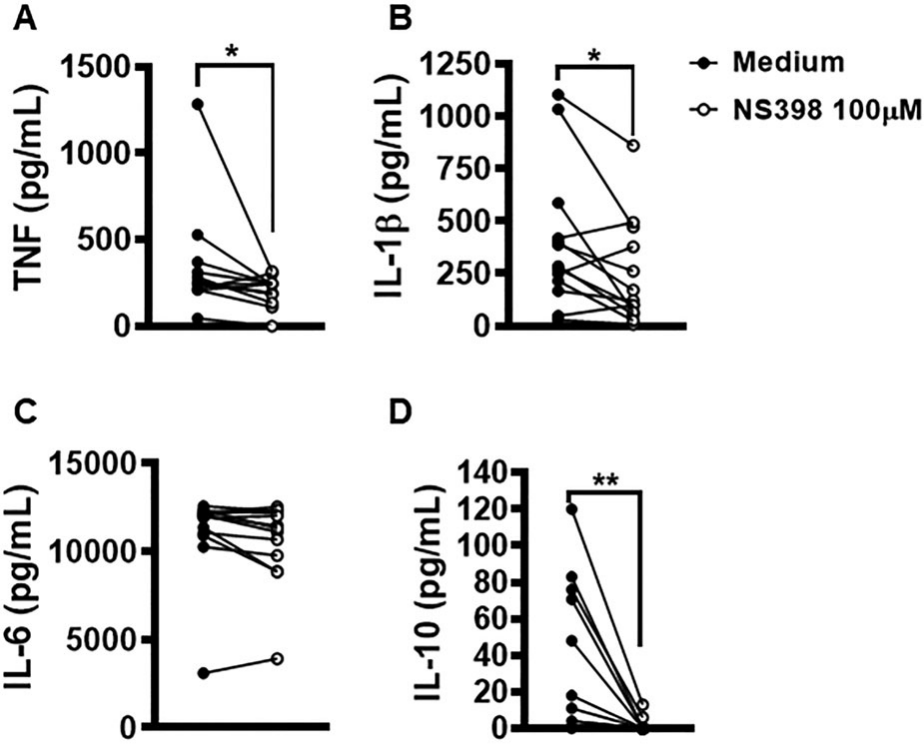
Neutralization of COX-2 in lesion cells decreases production of IL-1β, TNF and IL-10. Skin lesion biopsies from CL patients (*n* = 13) were obtained using a 4 mm punch and cultured for 72 h. Levels of (A) TNF, (B) IL-1β, (C) IL-6 and (D) IL-10 were determined in culture supernatants by ELISA. Statistical analysis performed using Wilcoxon's test; *P < 0.05 and **P < 0.005.

Lesions with high parasitic load exhibit a more intense inflammatory response observed to contribute to tissue damage and therapeutic failure [18]. NS398 is a COX-2 selective NSAID that was shown to be effective in controlling *L. infantum* infection *in vivo* [23]. Thus, we investigated whether the inhibition of COX-2 would exert any effect on macrophage infection by *L. braziliensis*. We found that COX-2 inhibition via NS398 decreased both the number of infected cells and the number of amastigotes at both 2 and 48 h after infection (Figure 7A,B). Regarding immune response, we observed that the treatment of cells residing in CL lesion sites with NS398 resulted in decreased TNF, IL-1β and IL-10 levels, yet no change in IL-6 was observed after 72 h of culture (Figure 8A–D), which suggests that other cells, i.e. not macrophages, are the producers of this cytokine. These results serve to indicate the benefits of neutralizing COX-2 in cells infected with *L. braziliensis*.

## Discussion

Increasing rates of failure to drug therapy have been observed in patients with CL over the years [4,5,24,25]. Host factors and parasite burden have been associated with the development of skin ulcers and failure to Glucantime® therapy in areas of high *L. braziliensis* transmission [18]. It follows that the identification of factors and mechanisms responsible for the disproportionate immune response observed in *Leishmania* infection, as well as those favouring the establishment of parasite persistence, remain extremely important. Lipid mediators are molecules with suspected involvement in resistance or susceptibility to pathogens in a range of infections [16,26–29]. PGE2, an inflammatory eicosanoid derived from the metabolism of arachidonic acid by COX enzymes, is known to contribute to the survival of various species of *Leishmania*, including *L. amazonensis*, *L. donovani* and *L. infantum* [17,20,23]. However, the roles played by COX-2 and PGE2 in CL immunopathogenesis arising from *L. braziliensis* infection have yet to be elucidated. The present study represents the first attempt to determine associations between COX-2 and PGE2 with respect to *L. braziliensis* survival, treatment failure and disease severity.

The activation of the EP2 receptor by PGE2 induces the production of cAMP, a molecule that decreases the production of oxygen derivatives [16,30,31], molecules relevant to the control of *Leishmania* proliferation [32]. It has been documented that increased COX-2 and EP2 gene expression in lesions from patients with cutaneous and diffuse leishmaniasis caused by *L. amazonensis* was associated with parasite survival [33]. Concordantly, we also identified increased expression of the genes encoding COX-2 and the EP2 receptor in the lesions of CL patients in comparison to healthy skin, which suggests that the COX-2/EP2 axis may be a crucial element in the survival and replication of *L. braziliensis*, as well as in therapeutic failure. Here, our results also show that, during the course of *L. braziliensis* infection*,* increased levels of COX-2 correlated positively with parasite load in the lesions of patients with CL. It is known that increased parasite load at the lesion site is associated with the uncontrolled production of a variety of inflammatory molecules (e.g. IL-1β) that contribute to tissue damage and, consequently, lead to therapeutic failure [6,18]. Our results additionally show that patients with high expression of the COX-2 gene failed therapy more frequently and presented greater numbers of lesions than those with low COX-2 gene expression. Moreover, we identified a strong correlation between the number of lesions and COX-2/PGE2 production, suggesting that increases in these molecules may be a contributing factor in disease severity. It is important to emphasize that patients with disseminated leishmaniasis, an emerging form of American Tegumentary Leishmaniasis (ATL), present multiple skin lesions and produce high levels of PGE2 [34]. Consequently, the data reported herein support the notion that exaggerated PGE2 production at lesion sites may be a key factor in parasite dissemination as well as the severity of disease.

The innate host immune response mediated by macrophages and neutrophils represents an important line of defence against parasites*,* due to the ability of these cells to produce ROS, molecules harmful to *Leishmania* spp. [8,27,32,35]. However, at the lesion sites of CL patients, low numbers of neutrophils in addition to a dense infiltrate containing macrophages have been documented [1,6]. Accordingly, we investigated the ability of macrophages to produce PGE2, as well as the role of this inflammatory eicosanoid in the context of *L. braziliensis* infection. It was previously demonstrated that macrophages from CL patients are capable of producing high levels of PGE2 in the first hours of infection by *L. braziliensis* [36]. Here we found that, following the internalization of *L. braziliensis* in macrophages, PGE production increased, reaching a peak at 48 h post-infection. We also observed that the levels of PGE2 at this timepoint positively correlated with the number of rounds of Glucantime®, which reinforces the involvement of this eicosanoid in therapeutic failure.

The inhibition of PGE2 synthesis through the use of NSAIDs, both those selective and non-selective for COX enzymes, has been shown to be effective in controlling infection by several species of *Leishmania in vitro* and *in vivo* [17,20,23]. However, it is important to emphasize that non-selective NSAIDs can interfere with pathways other than those involving COXs, and may provoke alterations in different physiological environments. Thus, our protocol employed the first selective NSAID described for COX-2 [37], which was applied to macrophages infected with *L. braziliensis* and cells from CL lesions. Our results indicate that the inhibition of COX-2 via NS398 decreased the numbers of amastigotes per macrophage, which stands in agreement with the findings from other studies involving other species of *Leishmania* and different NSAIDs [17,23]. An important result obtained in the current study was that although the neutralization of COX-2 in cells residing in CL lesions led to decreases in the secretion of cytokines TNF, IL-1β and IL-10, the addition of PGE2 in cultures of non-infected macrophages did not exert any effects on TNF, IL-1β and IL-6 production. To our surprise, we also found that exogenous PGE2 induced IL-10 production by non-infected macrophages. IL-10 is a well-described cytokine known to down modulate inflammatory responses [24,38], thus favouring parasite multiplication within macrophages [39]. However, the low concentration of IL-10 produced by infected macrophages is not capable of regulating the inflammatory response trigged during *L. braziliensis* infection. Even though PGE2 was able to increase IL-10 production in uninfected macrophages, the levels produced were apparently insufficient to regulate the inflammatory response observed during infection. Finally, we found that even at low concentrations IL-10 is capable of disabling the microbicidal functions of macrophages, thus favouring parasite proliferation. Together, these data show suggest that COX-2 enzyme inhibition using selective NSAIDs increases the ability of macrophages to kill *Leishmania*, thus attenuating inflammatory response.

In conclusion, the present study documents the advantages of inhibiting COX-2 with a selective NSAID, suggesting the role of NS398 as a promising candidate for adjuvant CL therapy.
